# Biological richness of a large urban cemetery in Berlin. Results of a multi-taxon approach

**DOI:** 10.3897/BDJ.4.e7057

**Published:** 2016-03-08

**Authors:** Sascha Buchholz, Theo Blick, Karsten Hannig, Ingo Kowarik, Andreas Lemke, Volker Otte, Jens Scharon, Axel Schönhofer, Tobias Teige, Moritz von der Lippe, Birgit Seitz

**Affiliations:** ‡ Department of Ecology, Technische Universität Berlin, 12165 Berlin, Germany; § Berlin-Brandenburg Institute of Advanced Biodiversity Research (BBIB), 14195 Berlin, Germany; | Callistus – Gemeinschaft für Zoologische & Ökologische Untersuchungen, 95503 Hummeltal, Germany; ¶ Senckenberg Research Institute, 60325 Frankfurt am Main, Germany; # Bismarckstr. 5, 45731 Waltrop, Germany; ¤ Senckenberg Museum of Natural History, 02826 Görlitz, Germany; « NABU Berlin, 13187 Berlin, Germany; » Deptartment of Evolutionary Biology, University of Mainz, 55128 Mainz, Germany

**Keywords:** bats, Berlin, birds, bryophytes carabids, harvestmen, graveyard, lichens, plants, spiders, urban cemetery

## Abstract

**Background:**

Urban green spaces can harbor a considerable species richness of plants and animals. A few studies on single species groups indicate important habitat functions of cemeteries, but this land use type is clearly understudied compared to parks. Such data are important as they (i) illustrate habitat functions of a specific, but ubiquitous urban land-use type and (ii) may serve as a basis for management approaches.

**New information:**

We sampled different groups of plants and animals in the Weißensee Jewish Cemetery in Berlin (WJC) which is one of the largest Jewish cemeteries in Europe. With a total of 608 species of plants and animals, this first multi-taxon survey revealed a considerable biological richness in the WJC. In all, 363 wild-growing vascular plant, 72 lichen and 26 bryophyte taxa were recorded. The sampling also yielded 34 bird and 5 bat species as well as 39 ground beetle, 5 harvestman and 64 spider species. Some species are new records for Berlin.

## Introduction

Cities can harbor a considerable number of plant and animal species (McKinney 2008, Shwartz et al. 2014). While there is a great deal of information on the biological richness of urban forest remnants and parks (e.g. Croci et al. 2008, Nielsen et al. 2014), cemeteries are clearly understudied, although this land-use type is ubiquitous in cities all over the world. Cemeteries are important components of the urban green infrastructure, simply because of their number and the area they cover. Berlin, for example, has 220 cemeteries within its limits, with a total area of about 1,125 hectares (SenStadtUm 2014).

A few studies on plants and animals, summarized in textbooks by Klausnitzer 1993 and Sukopp 1990 illustrate that cemeteries within a large city may play an important role for urban biodiversity due to their size, habitat heterogeneity and habitat continuity. Yet similarly to biodiversity studies on urban parks in general (Nielsen et al. 2014), earlier studies on the flora or fauna of cemeteries in Berlin have focused on single species groups and rarely comprise flora and fauna.

The present paper provides the first comprehensive inventory of a large urban cemetery in Berlin, the Weißensee Jewish cemetery (WJC), which is also one of the largest Jewish cemeteries (if not the largest) in Europe (Rütenik et al. 2013). The data set resulting from our multi-taxon approach includes information about the occurrences of several groups of plant taxa (vascular plants, bryophytes, lichens) and animal taxa (bats, birds, ground beetles, harvestmen, spiders). These data were recorded to support a World Heritage Initiative beginning in 2006, in particular to illustrate habitat functions of WJC that may back future approaches in linking biodiversity conservation and heritage preservation (Kowarik et al. 2011, Rütenik et al. 2013).

## Material and methods

### Study site

**Figure 1.**
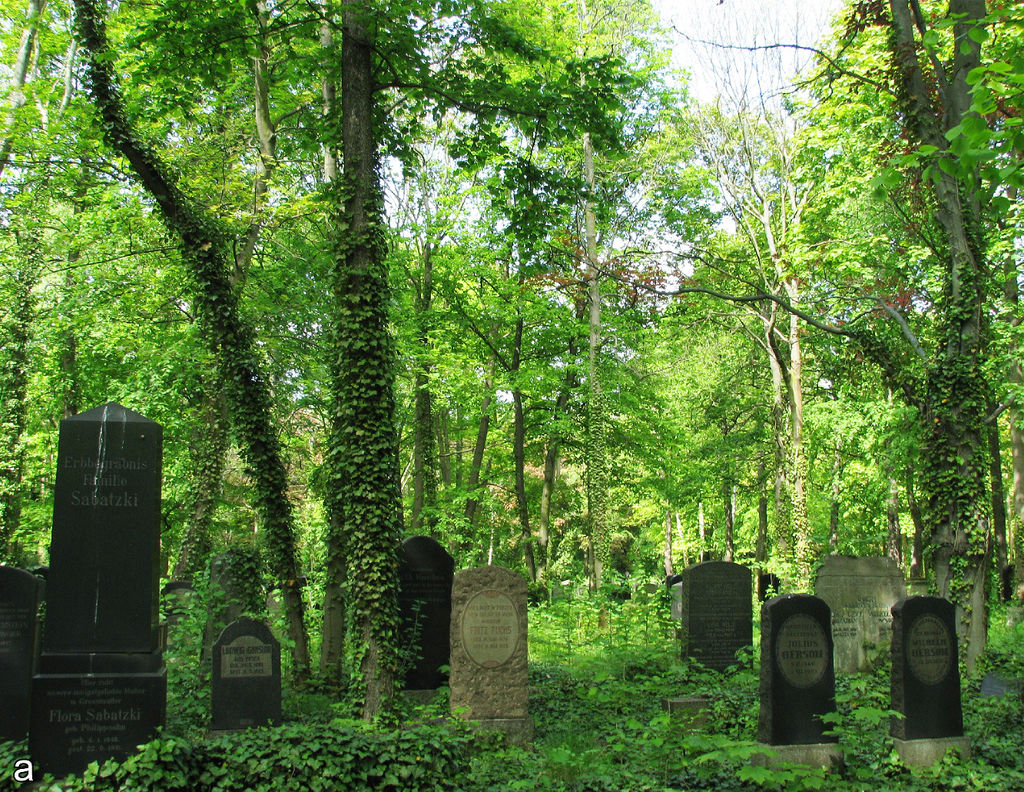
Impressions of the Weißensee Jewish Cemetery with regularly managed woodland. Photo: A. Lemke.

**Figure 2.**
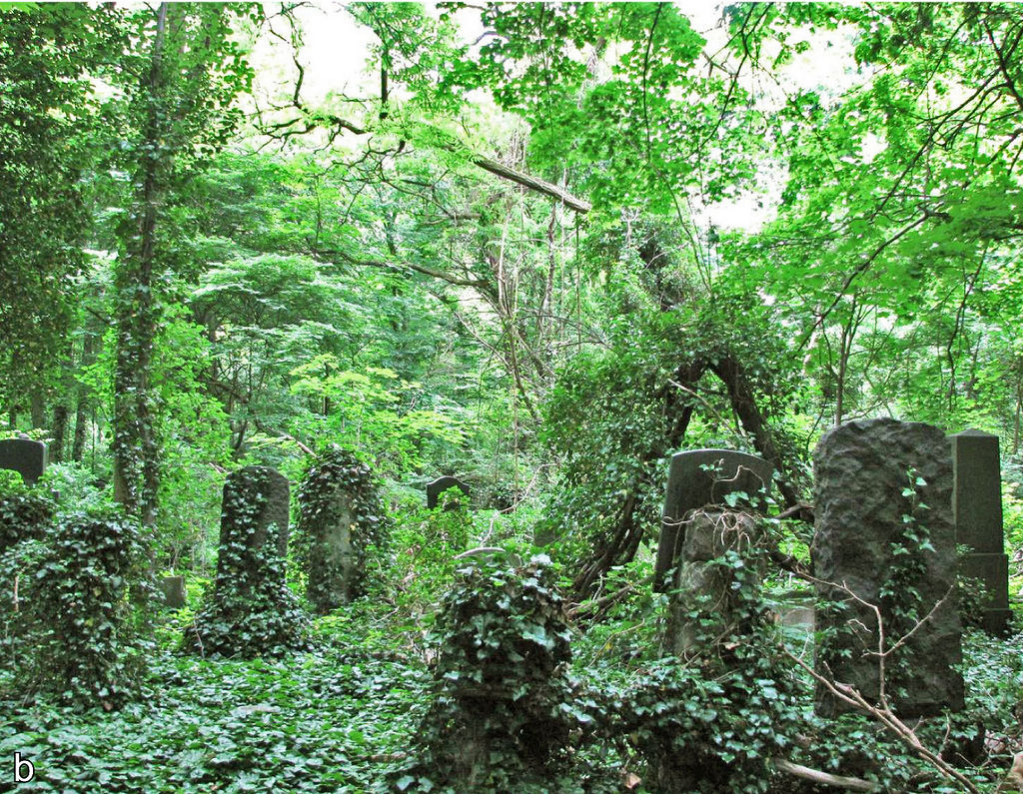
Impressions of the Weißensee Jewish Cemetery with irregularly managed woodland and parts of wildness. Photo: A. Lemke.

The study was performed in Berlin, Germany, which has a population of 3.5 million people within an area of 892 km^2^. The WJC is 39.2 ha and is situated in northeastern Berlin (central point: 52°32'40"N, 13°27'30"E). It was established in 1880 by the Jewish community and has about 116,000 graves (Rütenik et al. 2013). The design and plant use at WJC is similar to that of contemporary Christian cemeteries, with cemetery sections separated by tree-lined avenues (von der Lippe et al. 2011). The cemetery was never destroyed, but the numbers of burials and the intensity of management sharply declined at the end of the 1930s due to the Shoah (Rütenik et al. 2013). As a consequence, large parts of the WJC developed into woodland (Figs 1, 2). While only smaller parts of WJC, close to the entrance, were covered with frequently mowed lawns or with intensively managed ornamental plantings, major parts are dominated by trees. These woodlands are subject to different management intensities, ranging from regularly managed parts to unmanaged parts where wild woodlands were allowed to develop (Figs 1, 2). The main focus of management efforts is on uprooting wild tree saplings and shrubs. Vegetation structure and prevailing plant species are similar to other spontaneously grown woodlands in Central European cemeteries, with *Acer platanoides*, *A. pseudoplatanus* and *Fraxinus excelsior* as dominant tree species, and *Hedera helix*, *Dryopteris filix-mas* and *Impatiens parviflora* as abundant herb species (Passarge 1990). Features of the sepulchral architecture (types, materials, age), which shape many habitats for mosses, lichens and vascular plants, are extensively reported by Rütenik et al. 2013.

### Sampling methods

We sampled different groups of plants and animals in a nested design on three spatial scales (Table 1): an area-wide recording of the entire cemetery for bats, birds and vascular plants; a sampling of selected cemetery sections (n= 30) and selected family graves for lichens and bryophytes; and a sampling of 10 x 10 m plots (one in each of 30 selected cemetery section) for vascular plants, carabid beetles, harvestmen and spiders.

### Identification methods & analyses

For the multi-taxon survey, field observations and samplings were done by five experts (Table 2). Ground-dwelling arthropods caught in the traps were identified by three experts in the laboratory.

Based on the observed species richness of ground-dwelling arthropods we calculated the Chao 1 estimator using the R software environment (version 3.0.1, RCoreTeam 2013).

## Data resources

**Figure 3.**
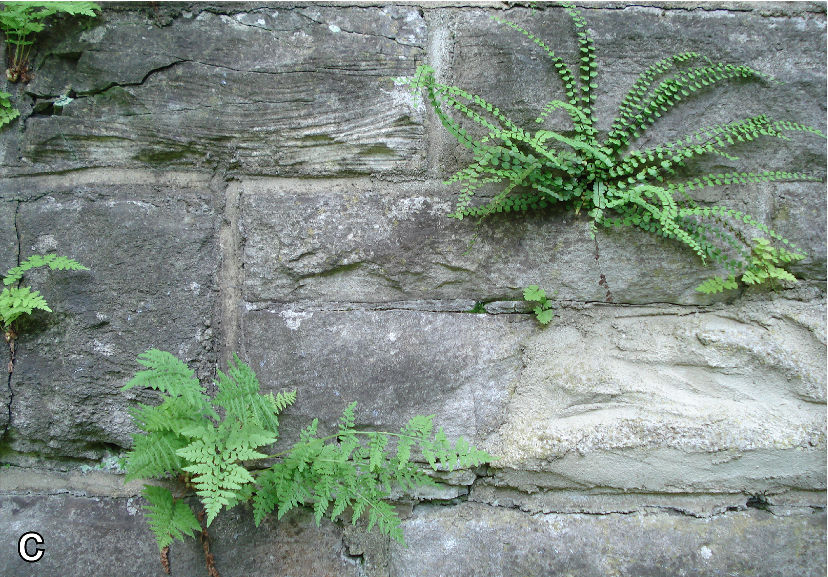
Examples for species with conservation concern: the ferns *Cystopteris fragilis*, *Asplenium trichomanes*. Photo: B. Seitz.

In all, 363 wild-growing vascular plant taxa were recorded. Of these, 140 grew within the sample plots (Table 3). Twenty-five plant taxa were of conservation concern (Fig. 3): 15 species were threatened, 5 species were near-threatened and 3 species, *Centaurium erythraea*, *Epipactis helleborine* and *Helichrysum arenarium*, were protected by law. Two species, *Potentilla sterilis* and *Urtica subinermis* were newly recorded for Berlin. *Urtica subinermis* was formerly assessed as a subspecies of *Urtica dioica* (Buttler and Hand 2007). The sampling of 30 cemetery sections revealed 72 taxa of lichens and 27 taxa of bryophytes (Tables 4, 5, Fig. 5). Two bryophytes and five lichens were threatened, one lichen species (*Hyperphyscia adglutinata*) was considered to be extinct in Berlin and one (*Aloxyria ochrocheila*) was newly recorded for Berlin and is very rare in northeastern Germany.

**Figure 4.**
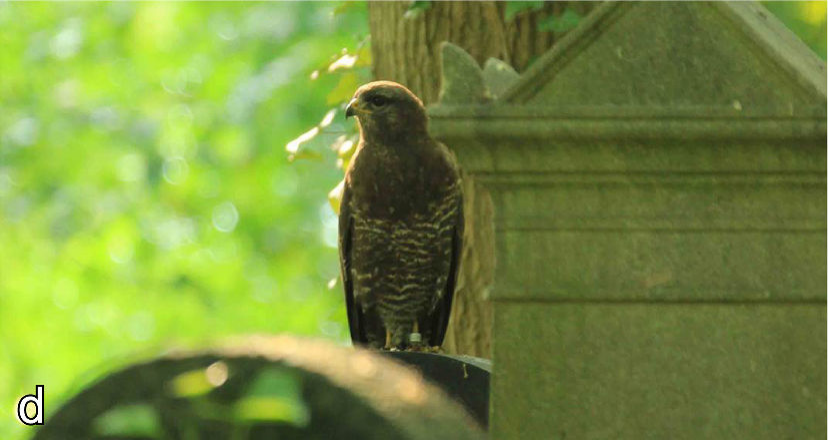
Examples for species with conservation concern: Common buzzard (*Buteo buteo*). Photo: J. Scharon.

**Figure 5.**
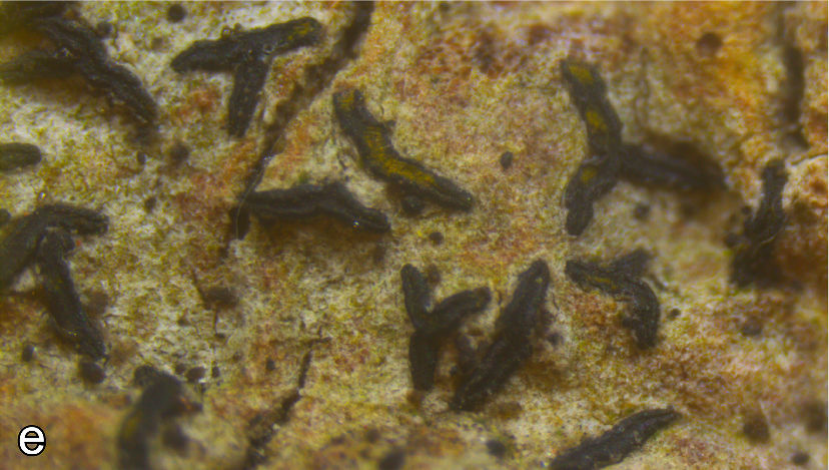
Examples for species with conservation concern: the lichen *Aloxyria ochrocheila*. Photo: V. Otte.

The area-wide recording yielded 34 bird and 5 bat species (Tables 6, 7). All bat species are listed in Annex IV of the Flora-Fauna-Habitat Directive and are thus of special conservation concern. Furthermore, nine of the bird species are protected (Fig. 4).

**Figure 6.**
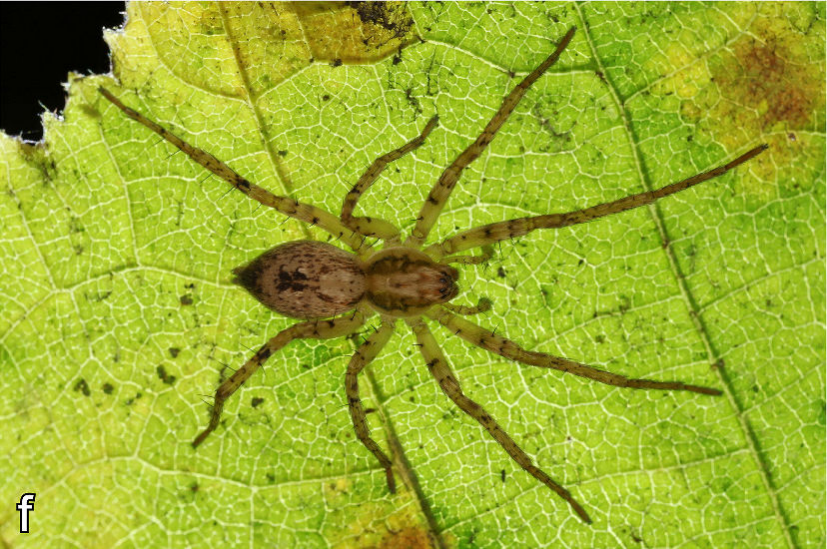
European spider of the year 2015: *Anyphaena accentuata*. Photo: C. Komposch.

During the plot-based sampling, 39 carabid beetle (793 individuals), 5 harvestmen (2247 individuals) and 64 spider (4559 individuals) species were caught (Table 8, Fig. 6). Among the ground-dwelling arthropods, two species were threatened and one, *Agonum gracilipes* (Carabidae), was considered to be extinct. *Nemastoma dentigerum* (Opiliones) and *Porrhomma microcavense* (Araneae) are new records for Berlin.

All species were native to Berlin except 118 species of vascular plants. The differentiation of natives and neophytes (post-1492 introductions) followed Seitz et al. 2012. Native species and pre-1492 introductions (i.e., archeophytes) were not differentiated.

### Conclusion

Our dataset illustrates that old cemeteries within a large city can harbor a considerable biological richness and therefore may play an important role for urban biodiversity conservation. Our results add evidence to findings from urban cemeteries and urban parks with large woodland patches (e.g., Graf 1986, Fudali 2001, Kocian et al. 2003, Croci et al. 2008, Gao et al. 2013, Philpott et al. 2014), but, for the first time, from a multi-taxon perspective. Our sampling approaches yield an overview over species assemblages of a range of groups of taxa. Yet due to limited sampling pressure, the inventories of the ground-dwelling arthropods are likely incomplete. Accordingly, species richness estimators show that a higher diversity can be expected, for example by applying other sampling techniques and catching for a larger timespan. However, although a two month sampling period is rather short, recent studies have shown that this period is sufficient to yield reliable data (Pearce et al. 2004, Vergnes et al. 2014). Pitfall traps only catch surface running species while species occurring in higher vegetation strata are rarely caught (Jimenez-Valverde and Lobo 2005). So further hand-sampling or sweep-netting studies should add a number of web building species to the species inventory presented. Since conservation and environmental planning is often restricted to few taxa (Gobbi et al. 2012), our dataset is especially important as it is the first which provides evidence that urban cemeteries could have an overall positive habitat function for many groups of taxa. Our data is thus a valuable snapshot that illustrates the relevance of this large urban cemetery for biodiversity conservation in Berlin. This is important information for urban planners, conservationists and gardeners that, however, needs being substantiated by further studies on the role of cultural parameters (e.g., management intensity) and environmental parameters (e.g., vegetation structure) in modulating biodiversity functions for different groups of taxa.

## Supplementary Material

Supplementary material 1 Location of sampled cemetery sections (grey) and sampled plots (small squares). Data type: Map
File: oo_79895.jpg Buchholz et al.

## Figures and Tables

**Table 1.** Sampling methods and spatial scales of the multi-taxon survey in the Weißensee Jewish Cemetery, Berlin.

**Taxon**	**Area-wide**	**Cemetery sections**	**Plots**
Bats	9 month period (January to September 2013) using bat detectors, mist-netting (one night in July 2013, 4 mist nets)	-	-
Birds	12 sampling days from April to June 2013 following Südbeck et al. 2005	-	-
Vascular plants	Repeated floristic inventories from April 2013 to October 2014	-	Vegetation inventories of 30 cemetery sections (10 x 10 m plots, April and May 2013) following Braun-Blanquet 1964
Lichens and bryophytes	-	Recorded in 30 cemetery sections and additionally on tombstones and other structures (e.g. walls, graves of honor); June to December 2013	-
Carabid beetles	-	-	Three pitfall traps (diameter 9 cm, depth 12 cm, filled with 4% formalin-detergent solution) per cemetery section (N = 30), April 24–June 24, 2013.
Harvestmen	-	-	see carabid beetles
Spiders	-	-	see carabid beetles

**Table 2.** Identification methods and experts of taxa sampled in the Weißensee Jewish Cemetery. Experts: AL = Andreas Lemke, AS = Axel Schönhofer, BS = Birgit Seitz, JS = Jens Scharon, KH = Karsten Hannig, TB = Theo Blick, TT = Tobias Teige, VO = Volker Otte

**Taxon**	**Identification**	**Nomenclature**	**Experts**
Bats	Dietz et al. 2007	Dietz et al. 2007	TT
Birds	Mullarney et al. 2011	Barthel and Helbig 2005	JS
Vascular plants	Jäger 2011	Buttler and Thieme 2015, Jäger 2011	BS, AL
Lichens and bryophytes	Nebel and Philippi 2005, Wirth et al. 2013	Nebel and Philippi 2005, Wirth et al. 2013	VO
Carabid beetles	Müller-Motzfeld 2006	Müller-Motzfeld 2006	KH
Harvestmen	Martens 1978	Martens 1978	AS
Spiders	Nentwig et al. 2015, Roberts 1987, Roberts 1998​	WSC 2015	TB

**Table 3.** Wild-growing vascular plants recorded during the area-wide and plot-based recording. Frequency was only assessed for species sampled in plots (100% = 30 plots). Explanations: Red List (Prasse et al. 2001): § = protected by law, * = not threatened, V = near threatened, 3 = vulnerable, 2 = endangered, 1 = critically endangered, ? = data deficient, nr = new record for Berlin; N = species non-native to Berlin, (N) = species native to Berlin with populations descending exclusively from cultivation (Seitz et al. 2012).

**Taxa**	**Frequency (%)**	**Red List / Protection**	**Non-native**
*Acer campestre*	10	R	.
*Acer platanoides*	93	*	(N)
*Acer pseudoplatanus*	83	*	N
*Achillea millefolium* s. l.	.	*	.
*Aegopodium podagraria*	10	*	.
*Aesculus hippocastanum*	10	*	N
*Agrostis capillaris*	.	*	.
*Agrostis gigantea*	.	*	.
*Agrostis stolonifera*	.	*	.
*Ailanthus altissima*	.	*	N
*Ajuga reptans* 'Atropurpurea'	3	*	N
*Alliaria petiolata*	10	*	.
*Allium vineale*	10	*	.
*Amaranthus retroflexus*	.	*	N
*Amelanchier lamarckii*		*	N
*Anemone blanda*	.	*	N
*Anemone nemorosa*	.	*	.
*Anthriscus sylvestris*	.	*	.
*Apera spica-venti*	.	*	.
Arabidopsis arenosa subsp. arenosa	.	*	.
*Arabidopsis thaliana*	7	*	.
*Arctium lappa*	.	*	.
Arenaria serpyllifolia subsp. serpyllifolia	7	*	.
*Arrhenatherum elatius*	3	*	N
*Artemisia campestris*	.	*	.
*Artemisia vulgaris*	3	*	.
*Asparagus officinalis*	.	*	.
*Asplenium ruta-muraria*	.	3	.
*Asplenium trichomanes*	.	2	.
*Atriplex patula*	.	*	.
*Atriplex sagittata*	.	*	.
Ballota nigra subsp. nigra	3	*	.
*Bellis perennis*	.	*	.
*Berberis thunbergii*	.	*	N
*Berberis vulgaris*	.	*	.
*Berteroa incana*	.	*	N
*Betula pendula*	17	*	.
*Bidens frondosa*	.	*	N
*Brachypodium sylvaticum*	.	*	.
*Bromus carinatus*	.	*	N
Bromus hordeaceus subsp. hordeaceus	.	*	.
*Bromus sterilis*	.	*	.
*Bromus tectorum*	.	*	.
*Bryonia dioica*	.	*	N
*Buddleja davidii*	.	*	N
*Buxus sempervirens*	10	*	N
*Calamagrostis epigejos*	7	*	.
*Campanula patula*	.	3	.
*Campanula persicifolia*	.	*	(N)
*Campanula rapunculoides*	.	*	.
*Capsella bursa-pastoris*	.	*	.
*Caragana arborescens*	.	*	N
*Cardamine hirsuta*	3	*	N
Carduus crispus subsp. crispus	.	*	.
*Carex acutiformis*	.	*	.
*Carex hirta*	.	*	.
Carex praecox subsp. praecox	3	*	.
*Carex spicata*	.	*	.
*Carex sylvatica*	.	*	(N)
*Centaurea stoebe*	.	*	.
*Centaurium erythraea*	.	2, §	.
*Cerastium arvense*	3	*	.
*Cerastium holosteoides*	.	*	.
*Cerastium semidecandrum*	3	*	.
*Cerastium tomentosum*	3	*	N
*Chaenorhinum minus*	.	*	N
*Chaerophyllum temulum*	7	*	.
*Chelidonium majus*	13	*	.
*Chenopodium album*	.	*	.
*Chenopodium hybridum*	.	*	.
*Chenopodium strictum* s. l.	.	*	N
*Chionodoxa luciliae x siehei*	3	*	N
*Chondrilla juncea*	.	*	.
*Cichorium intybus*	.	*	.
*Cirsium arvense*	.	*	.
*Cirsium vulgare*	.	*	.
*Clematis vitalba*	17	*	N
*Convallaria majalis*	3	*	.
*Convolvulus arvensis*	.	*	.
*Cornus mas*	.	*	N
*Cornus sanguinea*	3	*	.
*Corydalis solida*	.	*	N
*Corylus avellana*	3	*	.
*Corylus colurna*	7	*	N
*Corynephorus canescens*	.	*	.
Cotoneaster cf. horizontalis	.	*	N
*Crataegus monogyna*	3	*	.
*Crataegus monogyna* s. l.	.	*	.
*Crataegus x media*	.	1	.
*Crataegus x subsphaericea*	.	1	.
*Crepis capillaris*	3	*	.
*Crocus vernus* s. l.	.	*	N
*Cystopteris fragilis*	.	1	.
*Dactylis glomerata*	3	*	.
*Daucus carota*	.	*	.
*Deschampsia cespitosa*	.	*	.
*Descurainia sophia*	.	*	.
*Digitaria ischaemum*	.	*	.
*Draba verna*	7	*	.
*Dryopteris carthusiana*	3	*	.
*Dryopteris dilatata*	.	*	.
*Dryopteris filix-mas*	70	*	.
*Elaeagnus angustifolia*	.	*	N
Elymus repens subsp. repens	.	*	.
*Epilobium angustifolium*	.	*	.
*Epilobium ciliatum*	.	*	N
*Epilobium hirsutum*	.	*	.
*Epilobium montanum*	.	*	.
*Epilobium parviflorum*	.	*	.
*Epilobium lamyi*	3	*	N
*Epilobium tetragonum*	.	*	.
*Epipactis helleborine*	.	§	.
*Equisetum arvense*	7	*	.
*Eragrostis minor*	.	*	N
*Eranthis hyemalis*	.	*	N
*Erigeron annuus*	7	*	N
*Erigeron canadensis*	.	*	N
*Erysimum cheiranthoides*	.	*	.
*Euonymus europaea*	7	*	.
*Euphorbia cyparissias*	.	*	.
*Euphorbia peplus*	.	*	.
*Fagus sylvatica*	7	*	.
*Fallopia dumentorum*	.	*	.
*Festuca brevipila*	.	*	.
*Festuca pratensis*	3	*	.
*Festuca rubra*	.	*	.
*Ficaria verna*	7	*	.
*Filago arvensis*	.	1	.
*Fragaria ananassa*	.	*	N
*Fragaria vesca*	.	*	.
*Fraxinus excelsior*	70	*	.
*Gagea pratensis*	7	*	.
*Gagea villosa*	3	*	.
*Galanthus nivalis*	3	*	N
*Galanthus nivalis* 'Flore Pleno'	.	*	N
*Galeopsis tetrahit*	.	*	.
*Galinsoga parviflora*	.	*	N
*Galinsoga quadriradiata*	.	*	N
*Galium album*	.	*	.
*Galium aparine*	10	*	.
*Galium boreale*	.	3	.
*Galium x pomeranicum*	.	*	.
*Geranium pusillum*	.	*	.
*Geranium robertianum*	10	*	.
*Geum urbanum*	.	*	.
*Glechoma hederacea*	7	*	.
*Hedera helix*	100	*	(N)
*Helianthus annuus*	3	*	N
*Helichrysum arenarium*	.	§	.
*Heracleum sphondylium*	.	*	.
*Herniaria glabra*	.	*	.
*Hieracium aurantiacum*	.	*	N
*Hieracium lachenalii*	.	*	.
*Hieracium laevigatum*	.	*	.
*Hieracium murorum*	.	*	.
*Hieracium pilosella*	.	*	.
*Hieracium sabaudum*	.	*	.
*Holcus lanatus*	.	*	.
Hordeum murinum subsp. murinum	.	*	.
*Hosta* spec.	3	*	N
*Humulus lupulus*	23	*	.
*Hyacinthus orientalis*	.	*	N
*Hypericum perforatum*	3	*	.
*Hypochaeris radicata*	3	*	.
*Iberis sempervirens*	.	*	N
*Impatiens parviflora*	50	*	N
*Iris germanica*	.	*	N
*Juncus tenuis*	.	*	N
*Laburnum anagyroides*	3	*	N
*Lactuca serriola*	.	*	.
*Lamium album*	3	*	.
*Lamium amplexicaule*	.	*	.
*Lamium purpureum*	7	*	.
*Lapsana communis*	.	*	.
*Lathyrus latifolius*	.	*	N
*Lathyrus pratensis*	7	*	.
*Lepidium densiflorum*	.	*	N
*Lepidium ruderale*	.	*	.
*Lepidium virginicum*	.	*	N
*Leucanthemum ircutianum*	3	*	.
*Ligustrum vulgare*	17	*	N
*Linaria vulgaris*	.	*	.
*Lolium perenne*	.	*	.
*Lonicera tatarica*	.	*	N
*Lonicera xylosteum*	.	*	.
*Lotus pedunculatus*	.	*	.
*Luzula campestris*	.	*	.
*Luzula luzuloides*	.	*	N
*Luzula multiflora*	.	*	.
*Lychnis flos-cuculi*	.	3	.
*Lysimachia nummularia*	10	*	.
*Lysimachia vulgaris*	3	*	.
*Mahonia aquifolium*	37	*	N
*Matricaria discoidea*	.	*	N
*Matteuccia struthiopteris*	.	*	N
*Medicago lupulina*	3	*	.
*Medicago varia* s. l.	.	*	N
*Melica nutans*	.	V	.
*Melilotus albus*	.	*	.
*Moehringia trinervia*	3	*	.
*Muscari armeniacum*	.	*	N
*Mycelis muralis*	7	*	.
*Myosotis arvensis*	.	*	.
*Myosotis ramosissima*	7	*	.
*Myosotis sylvatica* s. l.	3	*	N
*Narcissus pseudonarcissus*	.	*	N
*Oenothera biennis* s. l.	.	*	N
*Oenothera pycnocarpa*	.	*	N
*Ornithogalum umbellatum* s. l.	.	*	N
*Oxalis dillenii*	.	*	N
*Oxalis stricta*	7	*	N
Papaver dubium subsp. dubium	.	*	.
*Parietaria pensylvanica*	.	*	N
*Parthenocissus quinquefolia*	.	*	N
*Parthenocissus tricuspidata*	.	*	N
*Persicaria maculosa*	.	*	.
*Phedimus spurius*	3	*	N
*Philadelphus coronarius*	.	*	N
*Phleum pratense*	3	*	.
*Picea abies*		*	N
*Picris hieracioides*	.	*	.
*Pinus sylvestris*	3	*	.
*Plantago lanceolata*	.	*	.
*Plantago major*	.	*	.
*Poa angustifolia*	.	*	.
*Poa annua*	7	*	.
*Poa compressa*	3	*	.
*Poa humilis*	.	*	.
*Poa nemoralis*	27	*	.
*Poa palustris*	3	*	.
*Poa pratensis*	3	*	.
Poa trivialis subsp. trivialis	.	*	.
*Polygonatum x hybridum*	3	*	N
*Polygonum aviculare*	.	*	.
*Populus nigra* 'Italica'	3	*	N
*Populus tremula*	.	*	.
*Portulaca oleracea*	.	*	N
*Potentilla argentea*	3	*	.
Potentilla recta subsp. recta	.	*	N
*Potentilla reptans*	.	*	.
*Potentilla sterilis*	.	nr	N
*Primula vulgaris*	.	*	N
*Prunella vulgaris*	.	*	.
*Prunus avium*	10	*	.
*Prunus cerasifera*	10	*	N
*Prunus domestica* s. l.		*	N
*Prunus padus*	13	*	.
*Prunus serotina*	.	*	N
*Pseudotsuga menziesii*	.	*	N
*Pulmonaria officinalis* s. l.	.	*	N
*Quercus petraea*	7	*	.
*Quercus robur*	17	*	.
*Quercus rubra*	3	*	N
Ranunculus acris subsp. acris	.	*	.
*Ranunculus auricomus* s. l.	3	3	.
*Ranunculus repens*	.	*	.
*Ranunculus sardous*	.	1	.
*Ribes alpinum*	10	*	N
*Ribes rubrum*	3	*	.
*Ribes uva-crispa*	23	*	N
*Robinia pseudoacacia*	3	*	N
*Rorippa palustris*	.	*	.
*Rosa canina*	13	*	.
*Rosa canina* s. l.	.	*	.
*Rosa corymbifera*	.	*	.
*Rubus armeniacus*	.	*	N
*Rubus caesius*	20	*	.
*Rubus idaeus*	3	*	.
*Rumex acetosa*	.	V	.
*Rumex acetosella*	3	*	.
*Rumex crispus*	.	*	.
*Rumex obtusifolius*	.	*	.
*Rumex thyrsiflorus*	3	*	.
*Sagina micropetala*	.	?	.
*Sagina procumbens*	7	*	.
*Salix alba*	.	*	.
*Salix caprea*	3	*	.
Salix cinerea subsp. cinerea	.	*	.
*Salix matsudana* 'Tortuosa'	.	*	N
*Salix viminalis*	.	*	.
*Salix x rubens*	.	*	.
*Sambucus nigra*	33	*	.
Sanguisorba minor subsp. balearica	.	*	N
*Saponaria officinalis*	.	*	.
*Saxifraga tridactylites*	3	3	.
*Scilla siberica*	40	*	N
Scorzoneroides autumnalis subsp. autumnalis	.	*	.
*Scrophularia nodosa*	.	*	.
*Securigera varia*	3	*	.
*Sedum acre*	3	*	.
*Sedum sexangulare*	.	*	.
*Sempervivum* spec.	3	*	N
*Senecio inaequidens*	.	*	N
*Senecio jacobaea*	.	*	.
*Senecio vernalis*	7	*	N
*Senecio viscosus*	.	*	.
*Senecio vulgaris*	3	*	.
*Setaria verticilliformis*	.	*	N
*Setaria viridis*	.	*	.
Silene latifolia subsp. alba	.	*	.
*Sisymbrium loeselii*	.	*	N
*Solanum decipiens*	.	*	N
*Solanum dulcamara*	.	*	.
*Solanum lycopersicum*	.	*	N
*Solidago canadensis*	7	*	N
*Solidago gigantea*	7	*	N
*Sonchus asper*	.	*	.
*Sonchus oleraceus*	.	*	.
*Sorbaria sorbifolia*	.	*	N
*Sorbus aucuparia*	7	*	.
*Sorbus intermedia*	3	*	N
*Spergularia rubra*	.	*	.
*Stellaria media*	3	*	.
*Stellaria pallida*	7	*	.
*Symphoricarpos albus*	3	*	N
*Symphyotrichum lanceolatum*	.	*	N
*Symphytum officinale*	.	*	.
*Syringa vulgaris*	13	*	N
*Tanacetum parthenium*	.	*	N
*Tanacetum vulgare*	3	*	.
Taraxacum cf. scanicum	.	*	.
Taraxacum Sect. Erythrosperma	3	*	.
Taraxacum Sect. Ruderalia	13	*	.
*Taxus baccata*	17	*	N
*Thuja occidentalis*	3	*	N
*Tilia cordata*	20	*	.
*Tilia platyphyllos*	3	*	N
*Tilia x vulgaris*	13	*	N
*Tradescantia virginiana*	.	*	N
*Trifolium arvense*	.	*	.
*Trifolium campestre*	.	*	.
*Trifolium dubium*	.	*	.
*Trifolium medium*	.	*	.
*Trifolium pratense*	3	*	.
*Trifolium repens*	.	*	.
*Tripleurospermum perforatum*	.	*	N
*Tsuga canadensis*	.	*	N
*Tulipa gesneriana* s. l.	3	*	N
*Tussilago farfara*	.	*	.
*Ulmus glabra* s. l.	57	V	.
*Ulmus laevis*	10	V	.
*Ulmus minor*	.	V	.
*Urtica dioica*	27	*	.
*Urtica subinermis*	.	nr	.
*Urtica urens*	.	*	.
*Verbascum nigrum*	.	*	.
Verbascum nigrum x cf. thapsus	.	*	.
*Verbascum thapsus*	.	*	.
*Veronica arvensis*	7	*	.
*Veronica chamaedrys*	10	*	.
*Veronica sublobata*	10	*	.
*Veronica officinalis*	.	*	.
*Veronica serpyllifolia*	3	*	.
*Viburnum opulus*	.	*	.
*Vicia angustifolia* s. l.	7	*	.
*Vicia cracca*	3	*	.
*Vicia hirsuta*	3	*	.
*Vinca minor*	20	*	N
*Viola odorata*	7	*	N
*Viola riviniana*	.	*	.
*Viola suavis*	7	*	N
*Viola x bavarica*	3	*	.
*Viola x wittrockiana*	.	*	N

**Table 4.** Lichens sampled on gravestones (g) and trees (t) in 30 cemetery sections and selected family graves. Frequency was only assessed for species sampled in cemetery sections (100% = 30 sections). Explanations: Red List (Otte 2005): * = not threatened, G = threat assumed, D = data deficient, 3 = vulnerable, 0 = extinct, ne = not evaluated), nr = new record for Berlin.

**Taxa**	**Frequency (%)**	**Red List**	**Habitat**
*Acarospo ramoenium*	.	*	G
*Aloxyria ochrocheila*	3	nr	t
*Amandinea punctata*	7	*	T
*Aspicilia contorta*	.	*	G
*Bacidina adastra*	3	ne	T
*Bacidina caligans*	3	ne	T
*Bacidina chloroticula*	3	D	T
*Bacidina neosquamulosa*	3	ne	T
*Bacidina* spec.	20	ne	G
*Buellia aethalea*	10	*	G
*Caloplaca chlorina*	67	D	t
*Caloplaca citrina*	27	*	g
*Caloplaca crenulatella*	10	*	g
*Caloplaca decipiens*	.	*	g
*Caloplaca flavocitrina*	30	*	g
*Caloplaca holocarpa*	7	*	g
*Caloplaca oasis*	7	ne	g
*Candelariella aurella*	27	*	g
*Candelariella reflexa*	7	*	t
*Candelariella vitellina*	10	*	g
*Cladonia chlorophaea*	7	*	t / g
*Cladonia coniocraea*	23	*	t
*Cladonia fimbriata*	10	*	t / g
*Clauzadea monticola*	3	ne	g
*Coenogonium pineti*	17	*	t
*Hyperphyscia adglutinata*	7	0	t
*Hypocenomyce scalaris*	3	*	t
*Hypogymnia physodes*	3	*	g
*Lecania cyrtella*	7	*	t
*Lecania naegelii*	3	D	t
*Lecanora albescens*	27	*	g
*Lecanora carpinea*	3	D	t
*Lecanora conizaeoides*	7	V	t / g
*Lecanora dispersa*	43	*	t / g
*Lecanora muralis*	17	*	t / g
*Lecanora persimilis*	7	ne	t
*Lecanora polytropa*	20	*	g
*Lecanora semipallida*	3	ne	g
*Lecidea fuscoatra*	7	3	g
*Lecidella scabra*	.	3	g
*Lecidella stigmatea*	50	*	g
*Leimonis erratica*	.	G	g
*Lepraria finkii*	70	*	g
*Lepraria incana*	80	*	t
*Parmelia sulcata*	20	*	t / g
*Phaeophyscia nigricans*	27	*	t / g
*Phaeophyscia orbicularis*	47	*	t / g
*Phlyctis argena*	3	3	t
*Physcia adscendens*	20	*	t / g
*Physcia tenella*	23	*	t / g
*Placynthiella dasaea*	3	D	t
*Placynthiella icmalea*	3	*	t
*Porina aenea*	60	*	t
*Porpidia soredizodes*	.	ne	g
*Pseudevernia furfuracea*	3	*	g
*Psilolechia lucida*	7	*	g
*Ramalina farinacea*	3	3	t
*Sarcogyne regularis*	.	*	g
*Scoliciosporum umbrinum*	.	*	g
*Stereocaulon vesuvianum*	.	G	g
*Trapelia coarctata*	7	*	g
*Trapelia obtegens*	.	*	g
*Trapelia placodioides*	.	*	g
*Verrucaria macrostoma*	3	ne	g
*Verrucaria muralis*	20	*	g
*Verrucaria nigrescens*	37	*	g
*Verrucaria tectorum*	3	ne	g
*Vezdaea aestivalis*	3	ne	g
*Vulpicida pinastri*	3	3	t
*Xanthoria candelaria*	3	*	t
*Xanthoria parietina*	13	*	t / g
*Xanthoria polycarpa*	10	*	t

**Table 5.** Bryophytes sampled in 30 cemetery sections (100% frequency = 30 cemetery sections). Explanations: Red List (Klawitter 2005): * = not threatened, 3 = vulnerable, 2 = endangered, ne = not evaluated.

**Taxa**	**Frequency (%)**	**Red List**
*Amblystegium serpens*	67	*
*Barbula unguiculata*	3	*
*Brachythecium albicans*	3	*
*Brachythecium rutabulum*	80	*
*Brachythecium salebrosum*	3	*
*Brachythecium populeum*	3	3
*Brachythecium velutinum*	13	*
*Bryoerythrophyllum recurvirostrum*	10	*
*Bryum argenteum*	10	*
*Bryum capillare*	67	*
*Ceratodon purpureus*	30	*
*Didymodon rigidulus*	27	*
*Eurhynchium hians*	23	*
*Grimmia pulvinata*	40	*
*Homalothecium sericeum*	3	2
*Hypnum cupressiforme*	33	*
*Hypnum lacunosum*	7	*
*Orthotrichum anomalum*	40	*
*Orthotrichum diaphanum*	27	*
*Plagiomnium cuspidatum*	3	*
*Polytrichum formosum*	3	*
*Rhynchostegium murale*	13	*
*Schistidium apocarpum* s. l.	37	*
*Schistidium apocarpum* s. str.	10	ne
*Schistidium crassipilum*	10	ne
*Tortula calcicolens*	7	ne
*Tortula muralis*	63	*

**Table 6.** Bat species recorded during the area-wide recording. Explanations: Red List (Altenkamp et al. 2005): 3 = vulnerable, 2 = endangered; conservation status: Annex IV (Flora-Fauna-Habitat Directive), records: bd = bat detector, o = observation, r = roosting site, pr = potential roosting site.

**Species**	**Red List**	**Conservation status**	**Record**
Common noctule (*Nyctalus noctula*)	3	Annex IV (FFH)	bd, o, r
Common pipistrelle (*Pipistrellus pipistrellus*)	3	Annex IV (FFH)	bd, o
Daubenton's bat (*Myotis daubentonii*)	2	Annex IV (FFH)	bd
Nathusius' pipistrelle (*Pipistrellus nathusii*)	3	Annex IV (FFH)	bd, pr
Serotine bat (*Eptesicus serotinus*)	3	Annex IV (FFH)	bd, o

**Table 7.** Bird species recorded during the area-wide recording. Explanations: Red List (Witt 2003): R = rare, V = nearly threatened, 3 = vulnerable, 2 = endangered; conservation status: § = protected, §§ = strictly protected.

**Species**	**Red List**	**Protection**	**Number of breeding territories**
Black bird (*Turdus merula*)	.	§	49
Blackcap (*Sylvia atricapilla*)	.	§	41
Blackstart (*Phoenicurus ochruros*)	.	§	1
Blue tit (*Parus caeruleus*)	.	§	10
Chaffinch (*Fringilla coelebs*)	.	§	21
Chiffchaff (*Phylloscopus collybita*)	.	§	14
Common buzzard (*Buteo buteo*)	.	§§	1
Common redstart (*Phoenicurus phoenicurus*)	.	§	5
Common serin (*Serinus serinus*)	V	§	1
Dunnock (*Prunella modularis*)	.	§	2
Eurasian jay (*Garrulus glandarius*)	.	§	>4
Eurasian wren (*Troglodytes troglodytes*)	.	§	27
European robin (*Erithacus rubecula*)	.	§.	14
Firecrest (*Regulus ignicapillus*)	.	§.	3
Garden warbler (*Sylvia borin*)	V	§	1
Goldfinch (*Carduelis carduelis*)	.	§	3
Goshawk (*Accipiter gentilis*)	R	§§	1
Great tit (*Parus major*)	.	§	21
Great woodpecker (*Dendrocopos major*)	.	§	8
Green woodpecker (*Picus viridis*)	V	§§	1
Greenfinch (*Carduelis chloris*)	.	§.	21
Grosbeak (*Coccothraustes coccothraustes*)	.	§	>7
Icterine warbler (*Hippolais icterina*)	V	§	4
Lesser pied woodpecker (*Dendrocopos minor*)	V	§	2
Lesser whitethroat (*Sylvia curruca*)	.	§	1
Long-tailed bushtit (*Aegithalos caudatus*)	.	§	2
Nuthatch (*Sitta europaea*)	.	§	8
Short-toed treecreeper (*Certhia brachydactyla*)	.	§	5
Song thrush (*Turdus philomelos*)	.	§	10
Spotted flycatcher (*Muscicapa striata*)	V	§	7
Starling (*Sturnus vulgaris*)	.	§	15
Wagtail (*Motacilla alba*)	V	§	1
Wood warbler (*Phylloscopus sibilatrix*)	.	§	1
Woodpigeon (*Columba palumbus*)	.	§	>6

**Table 8.** Ground-dwelling arthropods recorded during the plot-based sampling. Explanations: Frequency (100% = 30 plots), Red List (carabid beetles: Kielhorn 2005, spiders: Platen and Broen 2005): 2 = endangered, 0 = extinct, nr = new record for Berlin.

**Taxa**	**Frequency (%)**	**Red List**	**Individual sums**
**Carabid beetles**			
*Agonum gracilipes*	3	0	1
*Amara aenea*	3	*	1
*Amara anthobia*	3	*	1
*Amara convexior*	23	*	7
*Amara familiaris*	7	*	9
*Amara makolskii*	23	*	32
*Amara similata*	43	*	24
*Anchomenus dorsalis*	23	*	9
*Asaphidion flavipes*	17	*	15
*Badister bullatus*	23	*	13
*Badister lacertosus*	57	*	45
*Bembidion lampros*	10	*	5
*Bembidion properans*	10	*	3
*Bembidion quadrimaculatum*	3	*	1
*Carabus nemoralis*	90	*	245
*Clivina fossor*	3	*	2
*Dyschirius angustatus*	3	2	3
*Elaphrus cupreus*	3	*	1
*Harpalus distinguendus*	3	*	1
*Harpalus luteicornis*	3	*	1
*Harpalus pumilus*	3	*	1
*Harpalus rufipes*	13	*	5
*Harpalus signaticornis*	10	*	3
*Harpalus tardus*	13	*	7
*Leistus rufomarginatus*	23	*	57
*Licinus depressus*	7	*	2
*Loricera pilicornis*	23	*	7
*Nebria brevicollis*	17	*	6
*Notiophilus biguttatus*	43	*	31
*Notiophilus palustris*	63	*	59
*Notiophilus rufipes*	53	2	75
*Ophonus laticollis*	3	*	2
*Poecilus cupreus*	13	*	5
*Poecilus versicolor*	3	*	1
*Pterostichus nigrita*	3	*	1
*Pterostichus oblongopunctatus*	87	*	83
*Pterostichus rhaeticus*	10	*	3
*Pterostichus strenuus*	33	*	25
*Syntomus foveatus*	3	*	1
**Observed species**	**39**		
**Estimated species (Chao 1)**	**70**		
**Harvestmen**			
Nemastomatidae			
*Nemastoma dentigerum*	13	nr	11
*Nemastoma lugubre*	63	*	150
Phalangiidae			
*Odiellus spinosus*	13	*	12
*Rilaena triangularis*	100	*	1943
Trogulidae			
*Trogulus tricarinatus*	67	*	131
**Observed species**	5		
**Estimated species (Chao 1)**	5		
**Spiders**			
Agelenidae			
*Eratigena atrica*	3	*	1
*Tegenaria ferruginea*	10	*	3
*Tegenaria silvestris*	43	*	24
Anyphaenidae			
*Anyphaena accentuata*	7	*	2
Clubionidae			
*Clubiona comta*	3	*	1
*Clubiona terrestris*	60	*	39
Dictynidae			
*Cicurina cicur*	87	*	168
Dysderidae			
*Dysdera crocata*	10	*	3
*Harpactea rubicunda*	67	*	63
Gnaphosidae			
*Haplodrassus silvestris*	53	*	46
Hahniidae			
*Hahnia ononidum*	3	*	1
Linyphiidae			
*Anguliphantes angulipalpis*	33	*	33
*Araeoncus humilis*	3	*	1
*Bathyphantes parvulus*	60	*	293
*Centromerus pabulator*	3	*	1
*Centromerus sylvaticus*	23	*	7
*Ceratinella brevis*	47	*	40
*Dicymbium nigrum brevisetosum*	3	*	1
*Diplocephalus cristatus*	33	*	25
*Diplocephalus latifrons*	97	*	432
*Diplocephalus picinus*	100	*	939
*Diplostyla concolor*	77	*	186
*Entelecara acuminata*	3	*	1
*Erigone atra*	13	*	4
*Erigone dentipalpis*	3	*	1
*Erigonella hiemalis*	7	*	3
*Floronia bucculenta*	3	*	1
*Gonatium rubellum*	90	*	330
*Gongylidium rufipes*	3	*	5
*Linyphia hortensis*	97	*	104
*Linyphia triangularis*	3	*	1
*Micrargus herbigradus*	10	*	4
*Microneta viaria*	63	*	149
*Neriene clathrata*	93	*	99
*Palliduphantes pallidus*	33	*	25
*Porrhomma errans*	7	*	4
*Porrhomma microcavense*	7	nr	2
*Stemonyphantes lineatus*	23	*	12
*Tenuiphantes flavipes*	80	*	60
*Tenuiphantes tenebricola*	20	*	20
*Tenuiphantes tenuis*	3	*	1
*Troxochrus scabriculus*	3	*	1
*Walckenaeria acuminata*	63	*	63
*Walckenaeria atrotibialis*	67	*	63
*Walckenaeria cucullata*	3	*	1
*Walckenaeria furcillata*	10	*	4
Liocranidae			
*Agroeca brunnea*	97	*	150
*Liocranum rupicola*	3	*	1
Lycosidae			
*Pardosa saltans*	53	*	534
*Trochosa terricola*	70	*	93
Mimetidae			
*Ero furcata*	40	*	23
Miturgidae			
*Zora spinimana*	93	*	98
Pholcidae			
*Pholcus opilionoides*	3	*	1
Phrurolithidae			
*Phrurolithus festivus*	13	*	4
Salticidae			
*Ballus chalybeius*	7	*	2
Segestriidae			
*Segestria senoculata*	23	*	7
Theridiidae			
*Enoplognatha ovata*	30	*	10
*Episinus angulatus*	23	*	15
*Euryopis flavomaculata*	3	*	1
*Neottiura bimaculata*	13	*	4
*Robertus lividus*	27	*	40
Thomisidae			
*Ozyptila praticola*	93	*	305
*Xysticus lanio*	7	*	2
Zodariidae			
*Zodarion italicum*	7	*	2
**Observed species**	**64**		
**Estimated species (Chao 1)**	**81**		
