# Supplementary figures and images for: Biological richness of a large urban cemetery in Berlin. Results of a multi-taxon approach
Source: Biodivers Data J. 2016 Mar 8;(4):e7057. doi: 10.3897/BDJ.4.e7057 (PMC4822058; doi:10.3897/BDJ.4.e7057)

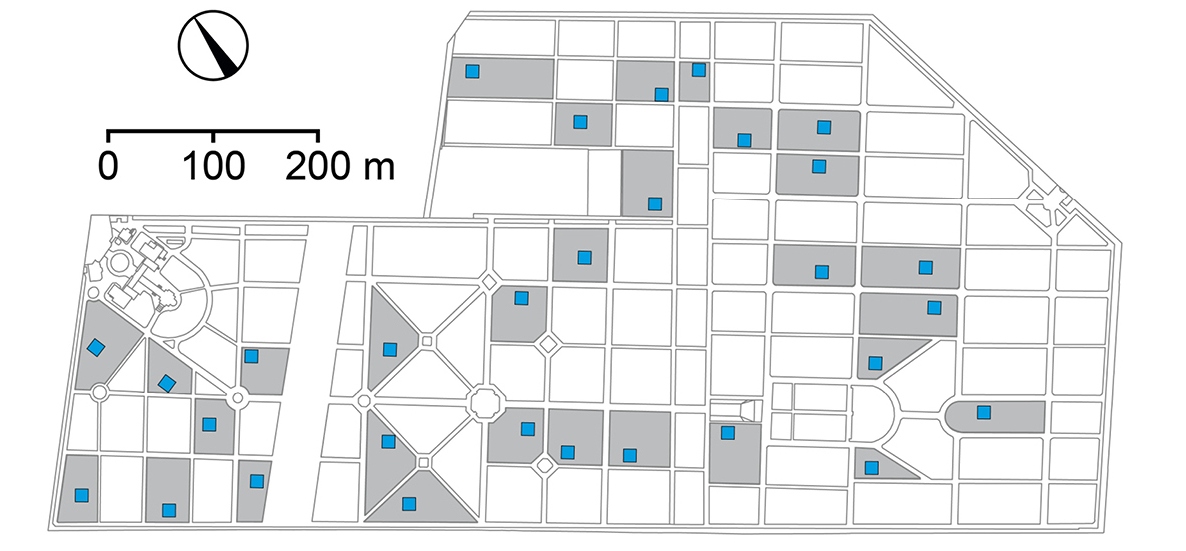

Supplement: Supplementary material 1 — Location of sampled cemetery sections (grey) and sampled plots (small squares). [file biodiversity_data_journal-4-e7057-s001.jpg]
